# Transcriptomic characterization of classical monocytes highlights the involvement of immuno-inflammation in bone erosion in Rheumatoid Arthritis

**DOI:** 10.3389/fimmu.2023.1251034

**Published:** 2023-10-05

**Authors:** Lucas Peixoto Sales, Bidossessi Wilfried Hounkpe, Mariana Ortega Perez, Valéria Falco Caparbo, Diogo Souza Domiciano, Eduardo Ferreira Borba, Georg Schett, Camille Pinto Figueiredo, Rosa Maria Rodrigues Pereira

**Affiliations:** ^1^ Rheumatology Division, Bone Metabolism Laboratory, Faculdade de Medicina, Universidade de São Paulo, São Paulo, Brazil; ^2^ Department of Internal Medicine 3-Rheumatology and Immunology, Friedrich Alexander Universität Erlangen-Nürnberg and Universitätsklinikum Erlangen, Erlangen, Germany

**Keywords:** rheumatoid arthritis, classical monocytes, bone erosions, inflammation, bone formation

## Abstract

**Introduction:**

Evidence-based data suggest that under inflammatory conditions, classical monocytes are the main source of osteoclasts and might be involved in bone erosion pathophysiology. Here, we analyze the transcriptomic profile of classical monocytes in erosive and non-erosive rheumatoid arthritis patients in order to better understand their contribution to bone erosion.

**Methods:**

Thirty-nine premenopausal RA patients were consecutively enrolled and divided into two groups based on the presence of bone erosions on hand joints. Classical monocytes were isolated from peripheral blood through negative selection, and RNA-seq was performed using a poly-A enrichment kit and Illumina® platform. Classical monocytes transcriptome from healthy age-matched women were also included to identify differentially expressed genes (DEGs). Therefore, gene sets analysis was performed to identify the enriched biological pathways.

**Results:**

RNA-seq analysis resulted in the identification of 1,140 DEGs of which 89 were up-regulated and 1,051 down-regulated in RA patients with bone erosion compared to those without bone erosions. Among up-regulated genes, there was a highlighted expression of *IL18RAP* and *KLF14* related to the production of pro-inflammatory cytokines, innate and adaptive immune response. Genes related to collagen metabolism (*LARP6*) and bone formation process (*PAPPA*) were down-regulated in RA patients with erosions. Enriched pathways in patients with erosions were associated with greater activation of immune activation, and inflammation. Interestingly, pathways associated with osteoblast differentiation and regulation of Wnt signaling were less activated in RA patients with erosions.

**Conclusion:**

These findings suggest that alterations in expression of monocyte genes related to the inflammatory process and impairment of bone formation might have an important role in the pathophysiology of bone erosions in RA patients.

## 1 Introduction

Rheumatoid arthritis (RA) is an autoimmune disease characterized by articular and extra-articular bone loss (1). Bone erosions, the hallmark lesions of RA, are induced by inflammation and are important predictor of disability, disease severity, and mortality in RA. Bone erosion pathophysiology involves the activation of immune cell clusters such as T cells, B cells, and monocytes/macrophages (2).

It has been demonstrated that classical monocytes subset represents about 80-90% of the total monocytes and secretes high levels of pro-inflammatory cytokines such as tumor necrosis factor alpha (TNF-α), interleukin-1 beta (IL-1β), and interleukin-6 (IL-6) in response to immune complex activation, and lipopolysaccharide stimulation (3). This subset also express the chemokine receptor CCR2 (4), which is related to transmigration of classical monocytes to inflammatory sites (5).

Considering that under inflammatory conditions, classical monocytes are an important source of pro-inflammatory cytokine production and are the main source of precursor of osteoclasts (6), these cells could represent an important link between inflammation and bone erosion in RA. Even in healthy individuals, classical monocytes express genes involved in immune responses, inflammatory process and tissue remodeling, such as *S100A12*, *S100A9*, *CSF3R*, and *MMP25* (7). Therefore, changes in gene expression of classical monocytes may substantially influence inflammation and bone loss in patients with RA.

Although evidence suggest that classical monocytes play a key role in supporting inflammation in RA patients, no data regarding the influence of these cells on bone destruction exists. Thus, the aim of the present study was to investigate the changes of the classical monocyte transcriptomic profile in erosive RA patients as compared to non-erosive patients.

## 2 Materials and methods

### 2.1 Study design and participants

Thirty-nine premenopausal RA women regularly followed in the RA Outpatient Clinic at the *Hospital das Clinicas da Universidade de São Paulo*, were recruited for this study. All patients fulfilled the American College of Rheumatology/European Alliance of Associations for Rheumatology 2010 classification criteria for RA. Patients were excluded if they presented metabolic bone disease (e.g., rickets, primary hypoparathyroidism, osteomalacia, and Paget’s disease); ii) bone-associated chronic disease (e.g., renal or liver failure, hyperthyroidism, hypothyroidism, and malabsorption); iii) use of any medication interfering with bone metabolism (e.g., prednisone doses higher than 7.5 mg/day, bisphosphonates, and bone-targeted monoclonal antibodies iv) an autoimmune disease other than RA, or v) being pregnant or lactating.

Premenopausal status was assessed through a self-reported information. Participants were asked to complete a questionnaire that included questions about their menstrual history, such as the age of menarche, regularity of menstrual cycles, and the presence or absence of menstrual periods in the past 12 months.

Based on data on bone erosion from high-resolution peripheral quantitative computed tomography (HR-pQCT), RA patients were classified into erosive and non-erosive patients according to the presence or absence of bone erosions on metacarpophalangeal (MCP) and proximal interphalangeal (PIP) joints of the dominant hand.

In addition, 20 age- and body mass index-matched healthy subjects were enrolled to the control group. The study was approved by the local Ethics Committee on Human Research from Sao Paulo University-CAPPesq (#51178115.1.0000.0068). All participants gave written informed consent, in accordance with the principles of the Declaration of Helsinki.

### 2.2 Clinical and laboratory assessments

Demographic and clinical data were obtained through interviews and medical records of the participants. Erythrocyte sedimentation rate (ESR), C-reactive protein (CRP), rheumatoid factor (RF), and anti–cyclic citrullinated peptide (anti-CCP) antibodies were measured using standard automated methods. Disease activity of RA patients was determined according to simplified disease activity index (SDAI), an activity disease score depicted as a simple linear sum of the follow parameters: tender and swollen joints count based on a 28-joint assessment, patient global assessment of disease activity (0–10), physician global assessment of disease activity (0–10) and CRP in mg/dL (8, 9).

### 2.3 Evaluation of bone erosions using HR-pQCT

Bone erosions were analyzed in RA patients using HR-pQCT as previously described (10). Briefly, HR-pQCT scans were performed in the second and third MCP and PIP joints of the dominant hand. Images were analyzed using OsiriX Lite (version 11.0.2; 32-bit for MacOS). Erosions were defined as a cortical break detected in two consecutive slices, visible in at least two planes, non-linear in shape, with underlying trabecular bone loss (11).

### 2.4 Classical monocytes isolation from human peripheral blood

Blood (70 ml) from RA patients and control group were collected, and peripheral blood mononuclear cells (PBMCs) were isolated by Ficoll density and dextran sedimentation. In both studied groups, monocytes were isolated from PBMCs by depletion of non-monocytes cells using an indirect magnetic labeling system. Classical monocytes were indirectly isolated using the CD16 MicroBeads human kit (Miltenyi Biotec®, Germany).

### 2.5 RNA‐sequencing

Classical monocytes RNA was isolated using the RNeasy Plus Mini (Qiagen®), and RNA integrity number (RIN ≥ 7) was evaluated by the ScreenTape method using Bioanalyser 2100 (Agilent®). RNA library was constructed using the Quant-seq® 30 mRNA-Seq Library Prep Kit (Lexogen®) and sequenced using an Illumina Seq 2500 platform (Illumina®).

### 2.6 RNA-sequencing data processing

Only high-quality raw sequencing data were included in the present study after trimming to remove low quality sequence and poly-A using BBDuk tool. Therefore, reads were mapped to human reference genome (GRCh38/Ensembl) using STAR aligner (12). Read count matrices were imported in R version 4.1 for downstream analyses.

### 2.7 Differential expression analysis

Raw read counts were pre-filtered and Differential Expression (DE) analysis based on negative binomial distribution was performed using DESeq2 and compared the transcriptome of RA patients with controls (13). In this first sub-analysis RA patient’s, data were merged in a unique disease group without considering their erosion status. RA patients were then classified in two subgroups based on the presence of erosion. Therefore, DE was performed using a likelihood ratio test (LRT) to compare differences between groups. Finally, a pairwise comparison was performed between the subsets of patients to identify differentially expressed genes (DEGs) in erosion patients group using Wald test (13). In both analyses, DEGs were identified based on fold change of 1.5 and a Benjamini-Hochberg false discovery rate (FDR) using a cut-off set at < 0.05.

### 2.8 Gene sets enrichment analysis and network reconstruction

Main biological processes associated with the top ranked DEGs were manually curated. Furthermore, more rigorous gene sets enrichment analyses were performed with GSEA and the single-sample GSEA (ssGSEA) tools (14). Threshold of p-value < 0.05 was considered significant. Network constituted by the significant GSEA pathways, and their inter-connected shared genes was reconstructed in Cystoscape (15). Network connectivity was analysed to identify hub genes, based on their centrality (highest degree).

List of relevant genes associated with RA was mined from Open target platform which integrates public data relevant to the association between targets and diseases including data from genetics, somatic mutations, expression analysis, drugs and animal models. The median Open Target association score and the centrality of the up-regulated genes were used to prioritize relevant targets in RA.

### 2.9 Correlation analysis

Pearson correlation analysis was performed to decipher the relationship between clinical and laboratorial characteristics, and pathway activity obtained from the GSEA score at single sample level (ssGSEA). p-value of < 0.05 were considered significant.

### 2.10 Volcano plot and heatmap

Volcano plots and Heatmaps were designed using ggplot2 package and the ComplexHeatmap package (16). Heatmap data were scaled by z-score and clustered using hierarchical clustering.

## 3 Results

### 3.1 Sample characteristics

The demographic and clinical characteristics of RA patients and control group are demonstrated in **Table 1**. All patients and controls were premenopausal women. Age, body mass index and disease activity were similar between erosive and non-erosive RA patients. Based on the SDAI scores, both the erosive and non-erosive patients demonstrated a moderate disease severity. The scores remained at 19.1 for erosive and 13.2 for non-erosive patients, showing no marked difference between them (p = 0.140). This moderate severity likely accounts for the consistent CRP levels seen across all groups. Notably, there was no significant variance in CRP levels when comparing either the erosive with non-erosive patients (p = 0.231) or the non-erosive patients with the controls (p = 0.090). In this way, CRP levels remained consistently comparable.

**Table 1:** Demographic and clinical characteristics.

Characteristic	Rheumatoid arthritis		Controls	P
	Erosive (n=26)	Non-erosive (n=13)	(n=20)	
Age, years	38.8 ± 7.1	36.6 ± 6.6	38.6 ± 6.8	0.635
BMI, kg/m^2^	26.4 ± 4.7	26.4 ± 5.4	25.7 ± 5.1	0.857
Race, No (%)				
White	17 (65.4)	8 (61.5)	15 (75)	0.296
Black	3 (11.5)	3 (23.1)	0 (0)	
Pardo¹	6 (23.1)	2 (15.4)	5 (25)	
ESR, mm/h	22.8 ± 26.1	8.9 ± 9.3	8.9 ± 7.1	0.005*^,$^
C-reactive protein, mg/L	3.4 (0.8-8.6)	2.2 (0.5-8.0)	1.1 (0.8-2.6)	0.111
Disease duration, years	8.0 (3.8-12.8)	7 (3.5-10.5)	–	0.501
Rheumatoid factor, n (%)	26 (100)	10 (76.9)	–	**0.031**
anti-CCP, n (%)	24 (92.3)	7 (53.8)	–	**0.010**
SDAI	19.1 (11.2-25.5)	13.2 (5.5-19.8)	–	0.140
Glucocorticoid, n (%)	12 (46.2)	7 (53.8)	–	0.651
Glucocorticoid, mg/day	5.0 (5.0-5.0)	5.0 (3.0-7.5)	–	0.606
csDMARD n (%)	25 (96.2)	11 (84.6)	–	0.253

Results are expressed in mean ± SD, median (IQR), or n (%). BMI, Body Mass Index; ESR, Erythrocyte Sedimentation Rate; anti-CCP, Anti-cyclic citrullinated peptide antibody; SDAI, Simplified Disease Activity Index; csDMARD, conventional synthetic Disease-modifying antirheumatic drugs. ¹Pardo is the exact term used in Brazilian Portuguese, meaning “mixed ethnicity”, according to the Brazilian Institute of Geography and Statistics. *erosive vs. non-erosive (P = 0.018); ^$^erosive vs. controls (P = 0.022). Bold values indicate statistically significant differences. The symbol "–" means that data is not available in the Controls.

The ESR was statistically significant between different groups as determined by one-way ANOVA. A Bonferroni *post hoc* test revealed that ESR was higher in erosive RA patients in comparison with non-erosive patients (p = 0.018), as well as with controls (p = 0.022) groups. The positivity for autoantibodies (RF and anti-CCP) was significantly higher in erosive RA patients in comparison to non-erosive patients.

### 3.2 Circulating classical monocytes exhibit an immuno-inflammatory phenotype in RA patients

**Figure 1:**
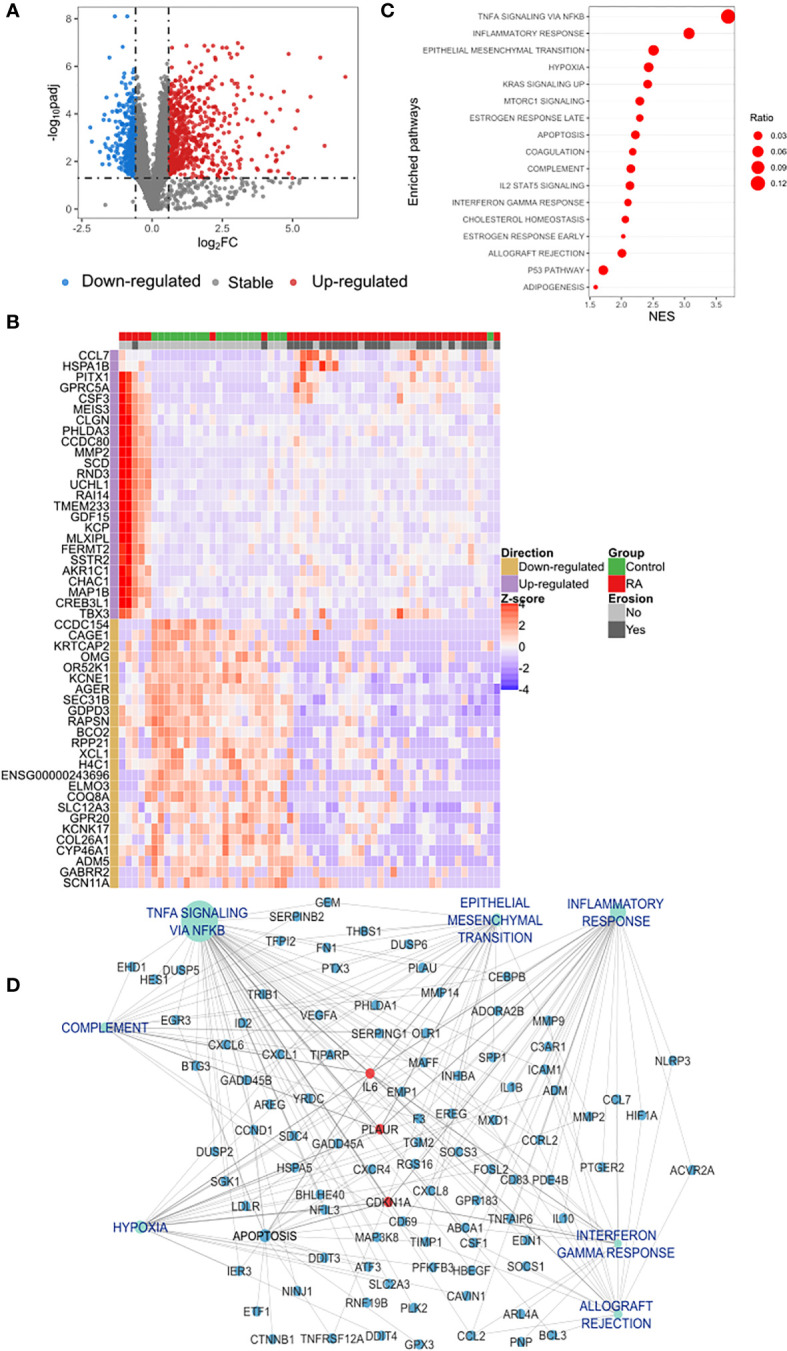
Differential expression analysis of classical monocytes’ genes in RA and functional gene set enrichment analysis **(A)** Protein coding transcriptome comparison of RA *vs.* control group showed a differential expression of 1,050 genes of which 693 were up-regulated (red dots) and 357 down-regulated (blue dots). Differentially expressed genes (DEGs) were identified based on a fold change of 1.5 and a Benjamini-Hochberg false discovery rate (FDR) using a cut-off set at < 0.05. **(B)** Unsupervised clustering of the expression pattern of the top-50 DEGs showed a clear stratification of RA and controls in two different clusters. Hierarchical clustering of samples was performed based on the Euclidean distance calculated from the normalized expression. **(C)** Dot plot of functional analysis performed using the hallmark gene sets showed the enrichment of biological processes associated with inflammation and immune activation. Dot size represents the ratio of DEGs that are involved each process and the X axis indicates the normalized GSEA enrichment score. Pathways with p-value of < 0.05 was considered significant. **(D)** Subnetwork of the GSEA showing the hub gene IL-6 connected with the most relevant pathways identified in the functional analysis. DEGs, Differential Expressed Genes; GSEA, Gene Set Enrichment Analysis; NES, Normalized Enrichment Score.

Gene expression profile of circulating classical monocytes of RA patients (including erosive and non-erosive RA patients) were compared with controls and showed 1,050 DEGs (**Figure 1A**) of which 693 were up-regulated and 357 down-regulated. Unsupervised clustering of samples using the expression pattern of the top-50 ranked genes (25 up- and 25 down-regulated) revealed a clear stratification of patients and control different clusters (**Figure 1B**).

Analyzing the list of DEGs, an enrichment of different families of cytokines and their receptors was observed in the up-regulated list of genes (**Supplementary Figure S1**). A manual curation of the main biological processes associated with the top 10 up-regulated genes also identified: the predominance of genes involved in inflammation and immune activation (*GDF15, CSF3, CCL7, MMP2*) as demonstrated in **Table 2**. In contrast, the main down-regulated genes were found in collagen formation (*COL26A1*) and bone mineralization (*CCDC154*) (**Table 2**).

**Table 2:** Top differentially expressed genes identified in rheumatoid arthritis patients (n=39).

Genes	Ave FC	FDR	Main biological process
Up-regulated genes			
GDF15	117.85	< 0.0001	Cytokine activity.
MMP2	70.62	0.0020	Degradation of the extracellular matrix.
CSF3	63.46	< 0.0001	Innate immunity.
UCHL1	49.84	< 0.0001	Ubiquitin-dependent protein catabolic process.
PITX1	36.24	< 0.0001	Cell growth and development.
CCDC80	34.32	0.0004	Cell adhesion and matrix assembly.
TMEM233	31.97	0.0490	Predicted to be integral component of membrane.
MLXIPL	29.12	0.0300	Carbohydrate and fatty acid homeostasis.
CCL7	29.02	< 0.0001	Innate Immunity.
AKR1C1	28.52	0.0020	Cholesterol metabolism.
Down-regulated genes			
COL26A1	0.22	0.0003	Collagen formation.
COQ8A	0.23	0.0010	Coenzyme Q10 biosynthesis and ATP production.
CAGE1	0.28	0.0039	Cancer testis antigens.
CCDC154	0.29	0.0100	Bone mineralization and maturation.
KCNK17	0.30	0.0001	Potassium transport.
BCO2	0.31	0.0001	Biosynthesis of vitamin A.
KRTCAP2	0.31	0.0018	Peptidyl-arginine modification.
SEC31B	0.32	< 0.0001	Unknown function.
OMG	0.32	< 0.0001	Development and correct function of central nervous system.
RPP21	0.34	0.0010	Ribonuclease P activity.

Genes were ranked according to the fold change. FC, Fold-change; Ave FC, average FC; FDR, False Discovery Rate.

### 3.3 Inflammatory response is driven by IL6, TNF and Interferon gamma in RA

To achieve further insights into the pathways that are increased in classical monocytes of RA as compared to controls, a functional gene set enrichment analysis, using all the DEGs identified in RA, was performed. This analysis further confirmed an increase in distinct biological processes associated with inflammation in RA (**Figure 1C**; **Supplementary Figure S2**). Furthermore, a network-based pathway was constructed (**Supplementary Figure S3A**) using the pathways shown in **Figure 1C** and their associated DEGs. Based on network centrality analysis (Degree), *IL6* was identified as the main hub gene, confirming its prominent role in the regulation of the inflammatory response observed in RA (**Supplementary Figure S3A**). The subnetwork containing only IL6 connections (**Figure 1D**) also contains two other top-ranked hub genes *CDKN1A, and PLAUR*, as well as central pathways such as TNF-a signaling via factor nuclear kappa B (NF-κB) (betweenness centrality 0.46 and 0.15 respectively). This indicates a coordinated regulation of these processes and suggests a proinflammatory network in classical monocytes in RA.

**Figure 2:**
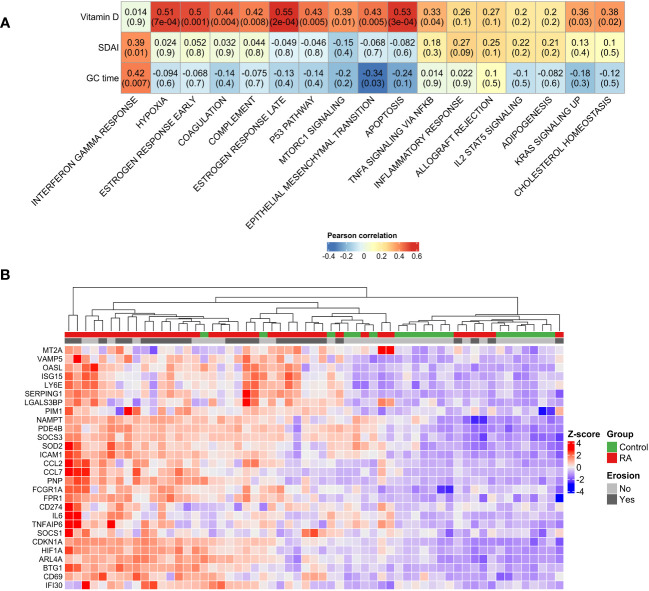
Disease manifestation and activated pathways. **(A)** Correlation plot showing the Pearson’s correlation between the GSEA pathways activated in RA and clinical parameters. High positive correlations have been observed between disease activity and interferon gamma pathway. Unexpectedly, positive correlations have been observed between vitamin D serum levels and TNF signaling, apoptosis and hypoxia. **(B)** Unsupervised clustering of interferon gamma response genes showed the stratification of RA patients and control group in two different groups. GC duration, Glucocorticoid duration; SDAI, Simplified Disease Activity Index; RA, Rheumatoid Arthritis.

Therefore, we investigated whether these pathways were also related to disease manifestation. Pearson’s correlation analysis revealed a correlation between the intensity of Interferon gamma response (ssGSEA score) and SDAI score (R = 0.39, p = 0.01) (**Figure 2A**). Unexpectedly, levels of vitamin D were positively correlated with hypoxia, early estrogen response, coagulation, late estrogen response and apoptosis pathways (**Figure 2A**). Furthermore, genes associated with interferon gamma response were sufficient to stratify RA patients and controls in two different clusters (**Figure 2B**), confirming its importance in RA pathogenesis.

### 3.4 Differential genes signature observed in bone erosion

**Figure 3:**
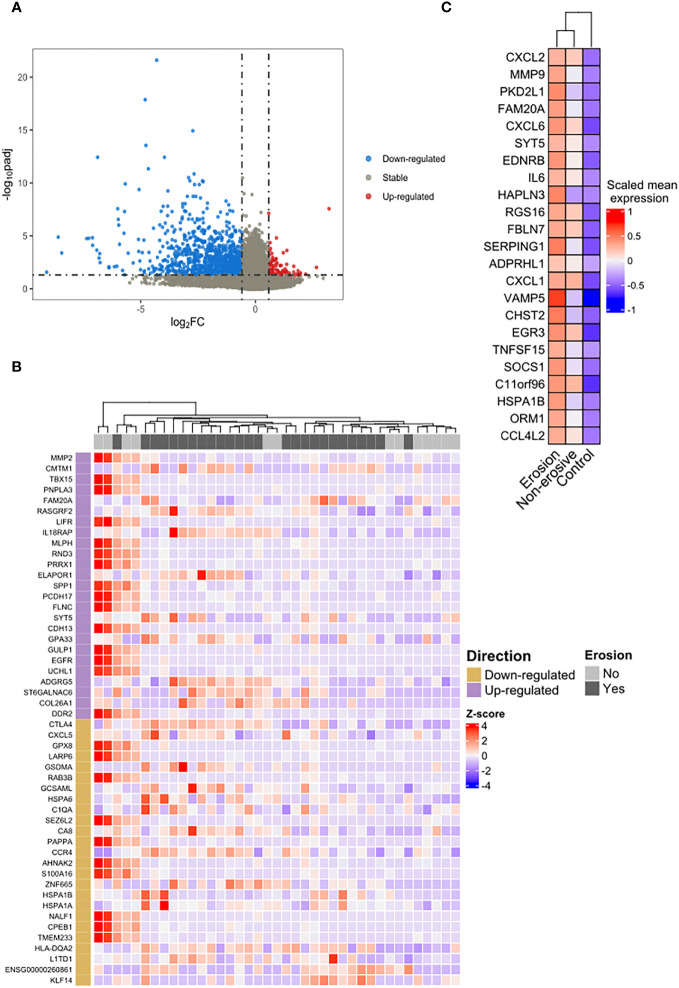
Differential expression and overrepresentation of immuno-inflammatory genes in bone erosion. **(A)** The comparison between RA patients with erosion and those without erosion identified 89 genes up-regulated (red dots) and 1,051 down-regulated (blue dots). **(B)** Heatmap of the top-50 highlighted a slight heterogeneity in the group of patients without erosion. **(C)** An overrepresentation of genes involved in cytokines response was identified in the list of shared up-regulated genes between the list of DEGs observed in erosion (erosion *vs*. without erosion) and those observed in RA regardless of the erosion status (RA *vs*. control).

In addition, we investigated the difference between the transcriptomic features of classical monocytes of RA patients considering their bone erosion status. A pairwise comparison (erosion *vs.* non- erosion) revealed 1,140 DEGs (**Figure 3A**) in RA patients with erosions, of which 89 were up-regulated and 1,051 down-regulated. To show the pattern of expression of the top ranked genes, a respective heatmap with the top-50 DEGs is presented in **Figure 3B**. Twenty-three genes that were up-regulated in RA regardless of erosion status (RA *vs.* controls) were also differentially expressed in patients with erosions in comparison with non-erosive patients (**Figure 3C**).

### 3.5 Bone erosion is driven by Immuno-inflammatory pathways

**Figure 3C** illustrates an enrichment of genes associated with immuno-inflammatory response such as IL6, CXCL1, CXCL2, CXCL6 and the metalloproteinase MMP9 in erosive disease. To verify whether this was a general characteristic of classical monocytes in erosive disease, we analyzed the biological processes that were enriched in the DEGs in erosive disease. A manual curation of the top ranked genes (**Table 3**) revealed immune response genes such as *HLA-DQA2, KLF14, IL18RAP, CMTM1* being upregulated in erosive disease. In the list of down-regulated genes, those associated with general biological and metabolic processes (**Table 3**) were identified. Interestingly, the down-regulated genes *PAPPA* and *AHNAK2* were associated with bone formation and wound healing (**Table 3**).

**Table 3:** Top differentially expressed genes identified in erosive rheumatoid arthritis patients (n=26).

Genes	Ave FC	FDR	Main biological process
Up-regulated genes			
*HSPA1B*	9.26	< 0.0001	Heat shock protein and ubiquitin-proteasome pathway.
*HLA-DQA2*	6.35	0.009525	Adaptive immunity.
*KLF14*	4.6	0.041606	Innate and adaptative immunity.
*IL18RAP*	3.9	0.031739	Cytokine activity.
*CMTM1*	3.83	0.043922	Chemokine activity.
*ST6GALNAC6*	3.76	0.043904	Sialyltransferase and alpha-N-acetylgalactosaminide alpha-2,6-sialyltransferase activity.
*ENSG00000260861*	3.67	0.049279	Unknown function.
*ZNF665*	3.64	0.019876	Regulation of transcription by RNA polymerase II.
*GSDMA*	3.54	0.039658	Apoptotic process.
*GPA33*	3.30	0.046824	Signaling receptor activity.
Down-regulated genes			
*NALF1*	0.0018	0.02	Calcium channel activity.
*LARP6*	0.0026	< 0.0001	Collagen biosynthetic.
*PRRX1*	0.0029	0.0004	Regulation of transcription by RNA polymerase II.
*RAB3B*	0.0061	< 0.0001	Regulation of ion transport.
*AHNAK2*	0.0065	< 0.0001	Wound healing.
*PAPPA*	0.0072	< 0.0001	Bone formation, inflammation and wound healing.
*RND3*	0.0073	< 0.0001	Cortical cytoskeleton organization.
*CPEB1*	0.0083	< 0.0001	Negative regulation of cytoplasmic translation.
*UCHL1*	0.0085	< 0.0001	Ubiquitin-dependent protein catabolic process.
*CDH13*	0.0089	0.001	Regulation of cell population proliferation.

Genes were ranked according to the fold change. FC, Fold-change; Ave FC, average FC; FDR, False Discovery Rate.

### 3.6 Increased activation of immuno-inflammation is correlated with disease activity and number of erosions

**Figure 4:**
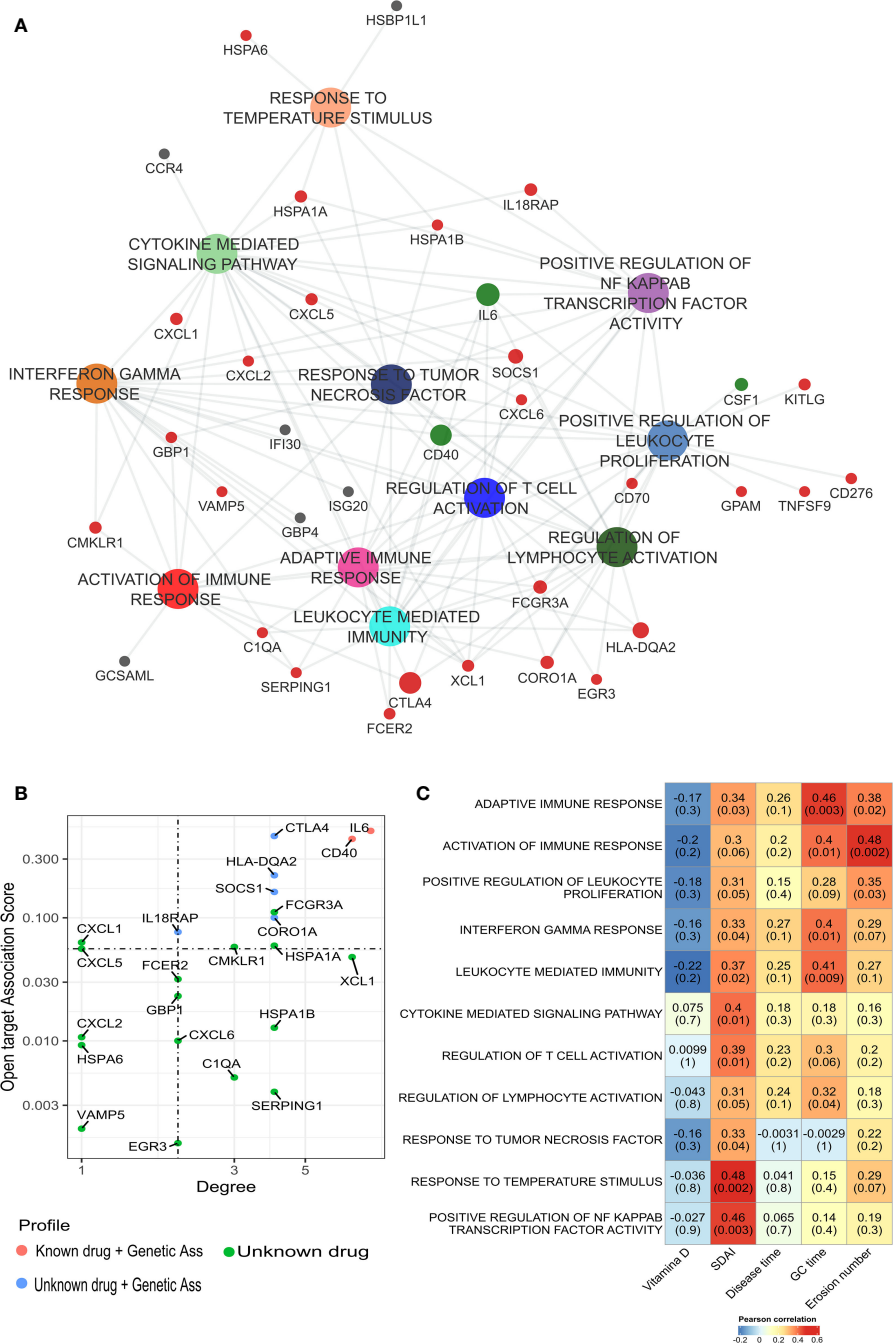
Gene set enrichment analysis of DEGs identified in classical monocytes from bone erosion. **(A)** ssGSEA analysis was performed to identified pathways that were enriched in erosion using the list of up-regulated genes. Enriched pathways are involved in immune-inflammatory processes with an overrepresentation of cytokine-mediated responses. **(B)** The network is enriched by genes associated with RA. Red dot: genes previously targeted by known drugs; blue: genes without drug but with genetic association. This information has been retrieved from Open Target database. **(C)** Pearson’s correlation analysis showed a positive correlation between bone erosion number and, cytokine and immune mediated pathway. Most of the pathways enriched in bone erosion are positively correlated with SDAI score. GC duration, Glucocorticoid duration; SDAI, Simplified Disease Activity Index; ssGSEA, Single Sample Gene Set Enrichment analysis.

The increase of immuno-inflammatory activation described above might potentially be a driver of bone erosion in RA. To test this hypothesis, we performed a gene set analysis using the DEGs identified in bone erosion and proceeded with a correlation analysis to identify any relationship between the biological pathways’ activation and disease activity. This gene sets enrichment analysis confirmed an increased activation of cytokine response genes, including response to interferon gamma and TNF, and the activation of immune response by leukocytes (**Figure 4A**). The network analysis identified *IL6, CD40, XCL1, SOCS1, CORO1A, HSPA1B, CTLA4, HLA-DQA2, HSPA1A, SERPING1* as the most connected genes with the highest degree of connectivity (ranging from 4 to 8). To further prioritize potentially relevant targets, we filtered the list of up-regulated genes identified in erosion with a list of relevant genes associated with RA downloaded from the Open target platform. This strategy identified *SOCS1, CORO1A, IL18RAP, CMKLR1 and HSPA1A* as genes associated with erosive RA (**Figure 4B**). These genes include most of the connected genes identified in the network analysis.

To identify the more activated pathways in bone erosion, a comparison of the activation rates on enriched pathways (ssGSEA score) between patients’ groups was performed. The most differentially activated pathways in classical monocytes in RA patients with bone erosions were related to immune response (**Supplementary Figures S4A–C**). In addition, a Receiver Operating Characteristic (ROC) curve analysis was performed, in order to evaluate the performance of these pathways to discriminate erosive RA from non-erosive RA based on their activation level (ssGSEA score). This analysis showed that these pathways might be applicable to classify erosion status, achieving an accuracy of at least 0.8 (**Supplementary Figures S5A–C**).

Moreover, it was checked whether the differential activation of these pathways was related to disease activity and erosions numbers by performing a Pearson’s correlation analysis. Interestingly, bone erosions number was positively correlated with the activity of immune pathways (**Figure 4C**), including activation of immune response (R = 0.48, p = 0.002), adaptative immune response (R = 0.38, p = 0.02), and positive regulation of leukocyte proliferation (R = 0.35, p = 0.03). It is worth mentioning that these pathways were those identified as suitable for distinguishing erosive from non-erosive disease (**Supplementary Figures S5A–C**). Furthermore, most of the enriched pathways related to bone erosions were positively correlated with SDAI score (**Figure 4C**).

### 3.7 CD14^+^CD16^-^ monocytes and down-regulation of osteoblast differentiation in bone erosion

**Figure 5:**
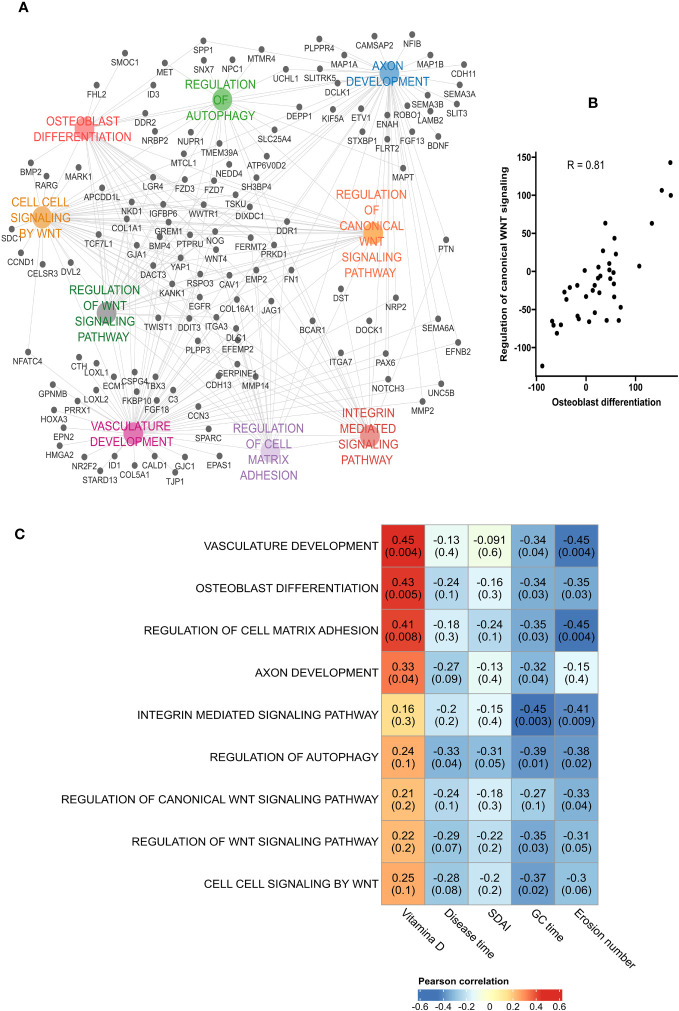
Down-regulation of osteoblast differentiation in bone erosion. **(A)** Pathways analysis of the down-regulated genes in erosion showed an enrichment of biological processes associated with osteoblast differentiation and bone formation. **(B)** High positive correlation was obtained between osteoblast differentiation and the regulation of canonical Wnt signaling. **(C)** Pearson’s correlation between the down-regulated pathways and patients’ characteristics showed a negative correlation between the number of erosion and pathways such as Vasculature development, Regulation of cell matrix adhesion, Regulation of autophagy and osteoblast differentiation. GC duration, Glucocorticoid duration; SDAI, Simplified Disease Activity Index.

We also investigated which biological pathways were down-regulated in erosive RA patients: Analyses found a predominance of pathways involved in osteoblast differentiation, and the regulation of canonical Wnt signaling pathway (**Figure 5A**). Hence correlation between the activation rate of osteoblast differentiation and the regulation of canonical Wnt signaling pathway (**Figure 5B**). Additionally, the most connected genes (*GREM1, NOG, DDIT3, COL1A1, PRKD1, LGR4, FERMT2, WNT4, RSPO3, WWTR1, CAV1*) were implicated in bone formation. When investigating the degree of down-regulation of these pathways and RA disease activity we found good correlation (**Figure 5C**). A negative correlation was observed between the number of erosions and the following pathways: Osteoblast differentiation (R = -0.35, p = 0.03), and Regulation of cell matrix adhesion (R = -0.45, p = 0.004).

## 4 Discussion

Herein we show the activation of classically activated monocytes in RA, which is even more pronounced in erosive than non-erosive disease. These data suggest that erosiveness in RA is linked to a pronounced activation of immune response genes in classical monocytes as well as a downregulation of genes that stimulate bone formation. Thus, alterations in the gene expression pattern in classically activated monocytes may orchestrate the altered bone homeostasis in RA.

Monocytes, i.e. classical monocytes have been associated with RA pathogenesis and disease progression (17). However, the association of specific monocytes subsets to bone erosion remained unclear. To the best of our knowledge, the implication of classical monocytes in bone erosions has not been studied at transcriptomic level in RA patients. When profiling the transcriptomic features of classical monocytes in RA patients, expression of immuno-inflammatory genes such as *IL6*, which are also known to stimulate osteoclast formation was higher in erosive than in non-erosive disease. Furthermore, a cluster of genes including *WNT4*, which are involved in bone formation, were down-regulated in monocytes from erosive RA patients.

Through the transcriptomic analysis this study also identified genes that were upregulated in classical monocytes of RA patients as compared to controls indicative for an upregulation of inflammatory and immune response genes in RA (18). In this way, among the top-ranked genes, we highlighted the upregulation of *GDF15*, CSF3, *CCL7* and *MMP2*.

The GDF15 gene encodes a secreted ligand of the TGF-beta (transforming growth factor-beta) superfamily of proteins (19). It is up-regulated in many pathological conditions, including inflammation (20). GDF-15 plays distinct roles in regulating biological processes such as bone formation and hematopoietic development (21). Moreover, it has garnered attention as a potential biomarker for several diseases and is considered an indicator of overall mortality risk (22).

CSF3 is a Protein Coding gene that encodes the granulocyte colony-stimulating factor 3. The encoded cytokine controls the production, differentiation, and function of granulocytes (19). In periodontal inflammation, suppressing *CSF3* has been shown to potentially reduce the mRNA expression of CXCL1, CXCL2 and CXCL3, IL-1β, IL-6 and matrix metalloproteinases 9 (23). Additionally, evidence suggests that a decrease in the receptor activator of nuclear factor κB ligand/osteoprotegerin (RANKL/OPG) ratio and the number of osteoclasts can contribute to the mitigation of damage to periodontal tissues (23). *CCL7* encodes the monocyte chemotactic protein 3 (MCP-3), a chemokine which attracts monocytes during inflammatory processes (24). Recently, it has been shown that CCL7 levels are higher in serum and synovial fluid of RA patients compared to patients with osteoarthritis (25). Moreover, CCL7 levels have been positively correlated with anti-CCP antibodies in synovial fluid (25), and have also been involved in promoting local bone resorption through osteoclast migrating and differentiation (26).

In the same way, MMP2, a member of the matrix metalloproteinase family, acts on the activation and cleavage of several chemokines, including CCL7, therefore contributing to the creation of a chemotactic gradient and subsequent immune cell infiltration (27). It has been demonstrated that MMP-2 and MMP-9 are expressed in synovial tissue by synoviocytes, vascular endothelial and monocytes derived macrophages, suggesting their contribution to angiogenesis and pannus formation in RA (28). These observations might indicate the capability of classical monocytes to modulate immune cell influx into synovial tissue and the remodeling of synovial tissue during inflammation.

When comparing gene expression of classical monocytes in erosive vs. non-erosive RA immune response genes and those related to cytokine activation were among the most differentially expressed ones. This finding is not surprising since several proinflammatory cytokines have osteoclastogenic properties and thereby contribute to bone erosion (29). In part, this cytokine upregulation can be explained by the observed up-regulation of *SOCS1* that is a negative-feedback inhibitor of JAK/STAT pathway (30). Of note, expression of cytokine-mediated pathways was also correlated with the extent of erosions.

Apart from cytokine pathways, some unexpected differences were observed among the expression patterns of monocytes from erosive and non-erosive RA patients. For instance, we found *KLF14*, a member of the Krüppel-like factors (KLF) family, as differentially expressed gene, which is involved in regulating T-regulatory cell differentiation (31). Furthermore, a recent study demonstrated that *KLF14* is down-regulated during osteogenic differentiation of bone marrow mesenchymal stem cells, and its overexpression suppressed cell viability and osteogenic differentiation (32). Interesting, expression of *KLF14* is negatively correlated with the *WNT3A* expression, a key factor for bone formation (32). The Wnt signaling is known to be implicated in the pathogenesis of many autoimmune diseases and vascular disorders and is one of the key biological processes associated with RA pathogenesis (33). Furthermore, Wnt pathway directly affects osteoblast and osteoclast, inducing an overall increase on bone formation and a decrease on bone resorption pathways (34).

Among the genes down-regulated in erosive RA are those associated with bone formation (*AHNAK2* and *PAPPA*). The family of protein encoded by *AHNAK2* plays a role in calcium signaling by interacting with calcium channel proteins, and an important role in bone metabolism (35). In fact, femur and tibia from *AHNAK* knock-out mice are shortened, and bone strength is significantly decreased (36). *PAPPA* gene, encodes a secreted metalloproteinase that cleaves insulin-like growth factor (IGF) binding proteins, and increases activation and availability of IGF (19). Inhibition of PAPPA in mice leads to reduced cortical area and trabecular thickness in long and vertebral bones (37). Furthermore, animals treated with anti-PAPP-A show impairment on bone formation rate and a tendency to reduce the osteoblast number, mineralizing surface, and osteoblast surface (37). Accordingly, pathways involved in osteoblast differentiation, and the regulation of canonical Wnt signaling pathway are down-regulated in erosive RA patients indicating that the myeloid compartment exerts negative effects on bone formation and repair.

In conclusion, the present study highlights the contribution of excessive activation of immuno-inflammatory pathways in classical monocytes as a contributing factor to bone erosion in patients with RA. Moreover, a downregulation of genes of bone formation processes was observed that suggest that classical monocytes orchestrate a milieu of suppression of bone formation and repair in erosive RA.

## Data availability statement

The original contributions presented in the study are publicly available. This data can be found here: https://www.ebi.ac.uk/biostudies/arrayexpress/studies/E-MTAB-13361.

## Ethics statement

The studies involving humans were approved by local Ethics Committee on Human Research from Sao Paulo University-CAPPesq (#51178115.1.0000.0068). The studies were conducted in accordance with the local legislation and institutional requirements. The participants provided their written informed consent to participate in this study.

## Author contributions

LS, VC, CF, and RP designed the research. LS, BH, MP, VC, DD, EN, CF, and RP performed the research. BH performed bioinformatics analysis. LS, BH, GS, CF, and RP wrote the manuscript, and MP, VC, DD, GS, and EN revised the manuscript. All authors contributed to the article and approved the submitted version.

## Glossary

**Table:**

AHNAK2	AHNAK Nucleoprotein 2
AKR1C1	Aldo-Keto Reductase Family 1 Member C1
anti-CCP	Anti–cyclic citrullinated peptide
BCO2	Beta-Carotene Oxygenase 2
CAGE1	Cancer Antigen 1
CAV1	CAV1
CCDC154	Coiled-Coil Domain Containing 154
CCDC80	Coiled-Coil Domain Containing 80
CCL7	C-C Motif Chemokine Ligand 7
CD40	CD40 Molecule
CDH13	Cadherin 13
CDKN1A	Cyclin Dependent Kinase Inhibitor 1A
CMKLR1	Chemerin Chemokine-Like Receptor 1
CMTM1	CKLF Like MARVEL Transmembrane Domain Containing 1
COL1A1	Collagen Type I Alpha 1 Chain
COL26A1	Collagen Type XXVI Alpha 1 Chain;COQ8A, Coenzyme Q8A
CORO1A	Coronin 1A
CPEB1	Cytoplasmic Polyadenylation Element Binding Protein 1
CRP	C-reactive protein
CSF3	Colony Stimulating Factor 3
CSF3R	Colony Stimulating Factor 3 Receptor
CTLA4	Cytotoxic T-Lymphocyte Associated Protein 4
DDIT3	DNA Damage Inducible Transcript 3
DE	Differential Expression
DEGs	Differentially expressed genes
ENSG00000260861	Novel Protein, SIRPB1-SIRPD Readthrough
ESR	Erythrocyte sedimentation rate
FDR	False discovery rate
FERMT2	FERM Domain Containing Kindlin 2
GDF15	Growth Differentiation Factor 15
GPA33	Glycoprotein A33
GREM1	Gremlin 1, DAN Family BMP Antagonist
GSDMA	Gasdermin A
GSEA	Gene sets enrichment analysis
HLA-DQA2	Major Histocompatibility Complex, Class II, DQ Alpha 2
HR-pQCT	High-resolution peripheral quantitative computed tomography
HSPA1A	Heat Shock Protein Family A (Hsp70) Member 1A
HSPA1B	Heat Shock Protein Family A (Hsp70) Member 1B
IGF	Insulin-like growth factor
IL18RAP	Interleukin 18 Receptor Accessory Protein
IL-1β	Interleukin-1 beta
IL6	Interleukin 6
IL-6	Interleukin-6
KCNK17	Potassium Two Pore Domain Channel Subfamily K Member 17
KLF	Krüppel-like factors
KLF14	KLF Transcription Factor 14
KRTCAP2	Keratinocyte Associated Protein 2
LARP6	La Ribonucleoprotein 6, Translational Regulator
LGR4	Leucine Rich Repeat Containing G Protein-Coupled Receptor 4
LRT	Likelihood ratio test
MCP	Metacarpophalangeal
MCP-3	Monocyte chemotactic protein 3
MLXIPL	MLX Interacting Protein Like
MMP2	Matrix Metallopeptidase 2
MMP25	Matrix Metallopeptidase 25
NALF1	NALCN Channel Auxiliary Factor 1
NF-κB	factor nuclear kappa B
NOG	Noggin
OMG	Oligodendrocyte Myelin Glycoprotein
PAPPA	Pappalysin 1
PBMCs	Peripheral blood mononuclear cells
PIP	Proximal interphalangeal
PITX1	Paired Like Homeodomain 1
PLAUR	Plasminogen Activator, Urokinase Receptor
PRKD1	Protein Kinase D1
PRRX1	Paired Related Homeobox 1
RA	Rheumatoid arthritis
RAB3B	RAB3B, Member RAS Oncogene Family
RF	Rheumatoid factor
RND3	Rho Family GTPase 3
ROC	Receiver operating characteristic
RPP21	Ribonuclease P/MRP Subunit P21
RSPO3	R-Spondin 3
S100A12	S100 Calcium Binding Protein A12
S100A9	S100 Calcium Binding Protein A9
SDAI	Simplified disease activity index
SEC31B	SEC31 Homolog B, COPII Coat Complex Component
SERPING1	Serpin Family G Member 1
SOCS1	Suppressor of Cytokine Signaling 1
ssGSEA	single-sample GSEA
ST6GALNAC6	ST6 N-Acetylgalactosaminide Alpha-2,6-Sialyltransferase 6
TMEM233	Transmembrane Protein 233
TNF-α	Tumor necrosis factor alpha
UCHL1	Ubiquitin C-Terminal Hydrolase L1
WNT3A	Wnt Family Member 3A
WNT4	Wnt Family Member 4
WWTR1	WW Domain Containing Transcription Regulator 1
XCL1	X-C Motif Chemokine Ligand 1
ZNF665	Zinc Finger Protein 665
