# Supplementary material for: Transcriptomic characterization of classical monocytes highlights the involvement of immuno-inflammation in bone erosion in Rheumatoid Arthritis
Source: Front Immunol. 2023 Oct 5;14:1251034. doi: 10.3389/fimmu.2023.1251034 (PMC10588645; doi:10.3389/fimmu.2023.1251034)

**Supplementary Material**

**Transcriptomic characterization of classical monocytes highlights the involvement of immuno-inflammation in bone erosion in Rheumatoid Arthritis.**

Lucas Peixoto Sales¹*, Bidossessi Wilfried Hounkpe¹*, Mariana Ortega Perez¹, Valéria Falco Caparbo¹, Diogo Souza Domiciano¹, Eduardo Ferreira Borba¹, Georg Schett², Camille Pinto Figueiredo¹#, Rosa Maria Rodrigues Pereira¹$

*** Equal contribution; $ *in memoriam***

**Supplementary Figure S1.** Differentially expressed cytokines and their receptors in Rheumatoid Arthritis.

**Supplementary Figure S2.** Differentially activated gene set enrichment analysis showing once again a stratification of Rheumatoid Arthritis patients and healthy controls in two different clusters.

**Supplementary Figure S3.** Gene set enrichment network. Connected network of the pathways enriched in Rheumatoid Arthritis. Pathways are interconnected by theirs shared genes. The network connectivity analysis identified IL6, PLAUR and CDKN1A (highlighted in red) as the most connected genes. The size of each dot indicates the importance of the represented pathways in this network. The fold change of each gene is indicated by the intensity of coloration of the small dots. Network is reconstructed in Cytoscape based on GSEA analysis.

**Supplementary Figure S4.** Highly activated pathways in classical monocytes of bone erosion patients. The most differentially activated pathways are involved in the activation of immune response (A); the positive regulation of leukocyte proliferation (B); and the adaptative immune response (C). ssGSEA scores were compared using Wilcoxon Rank Sum Test. No: patients without erosion; Yes: patients with bone erosion; ssGSEA: single sample Gene Set Enrichment Analysis.

**Supplementary Figure S5.** Classifiers of bone erosion. Using pathway activity at single sample level (ssGSEA) and ROC curve analysis, 3 pathways were identified as good classifiers of bone erosion. These includes: (A) Activation of immune response; (B) Positive regulation of leukocyte proliferation; (C) and the adaptative immune response. ssGSEA: Single Sample Gene Set Enrichment analysis; ROC: Receiver Operating Characteristic.

**Supplementary Figure S1.** Differentially expressed cytokines and their receptors in Rheumatoid Arthritis.


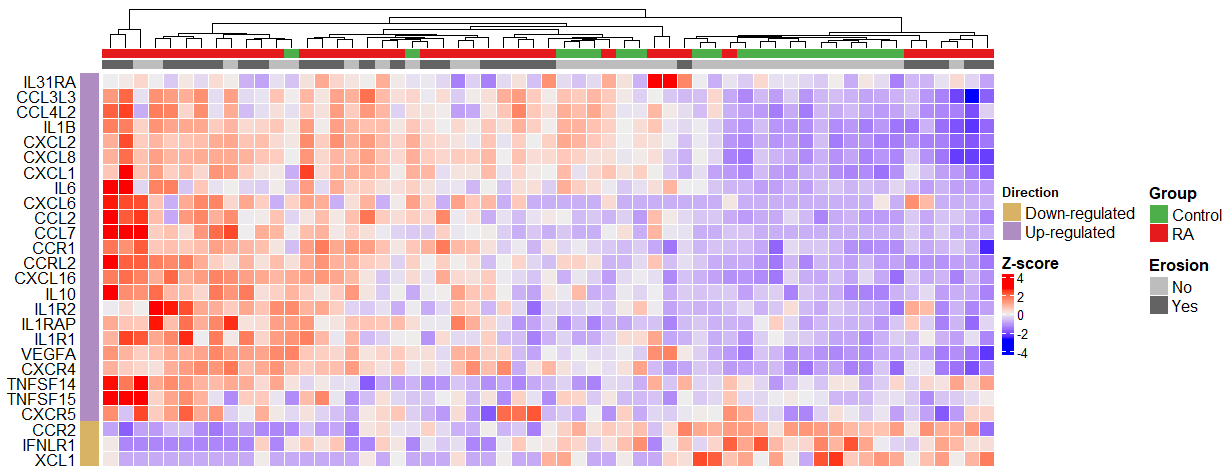


**Supplementary Figure S2.** Differentially activated gene set enrichment analysis showing once again a stratification of Rheumatoid Arthritis patients and healthy controls in two different clusters.


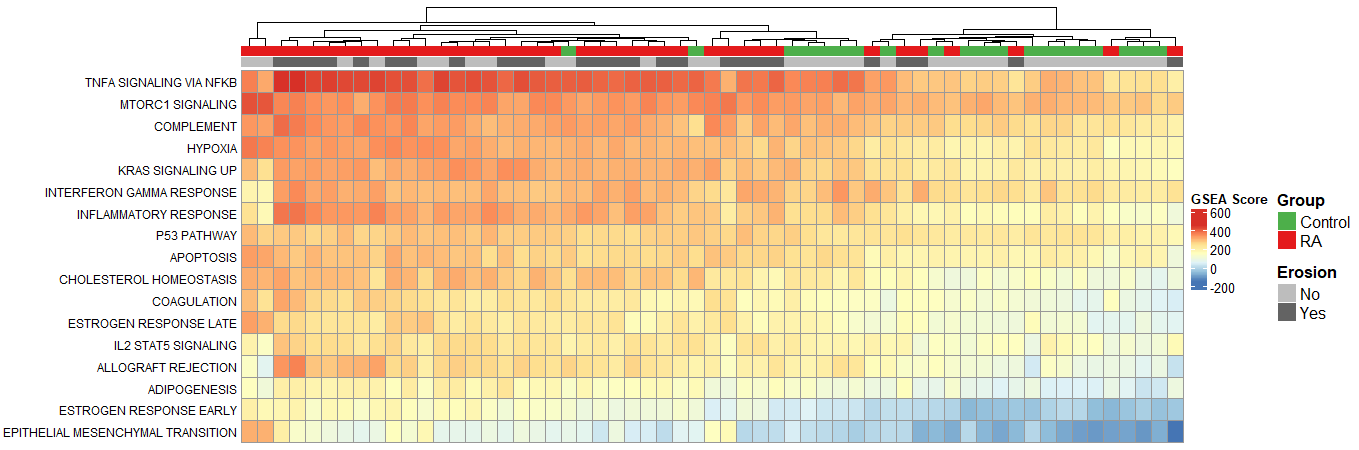


**Supplementary Figure S3.** Gene set enrichment network. Connected network of the pathways enriched in Rheumatoid Arthritis. Pathways are interconnected by theirs shared genes. The network connectivity analysis identified IL6, PLAUR and CDKN1A as the most connected genes. The size of each dot indicates the importance of the represented pathways in this network. The fold change of each gene is indicated by the intensity of coloration of the small dots. Network is reconstructed in Cytoscape based on GSEA analysis.


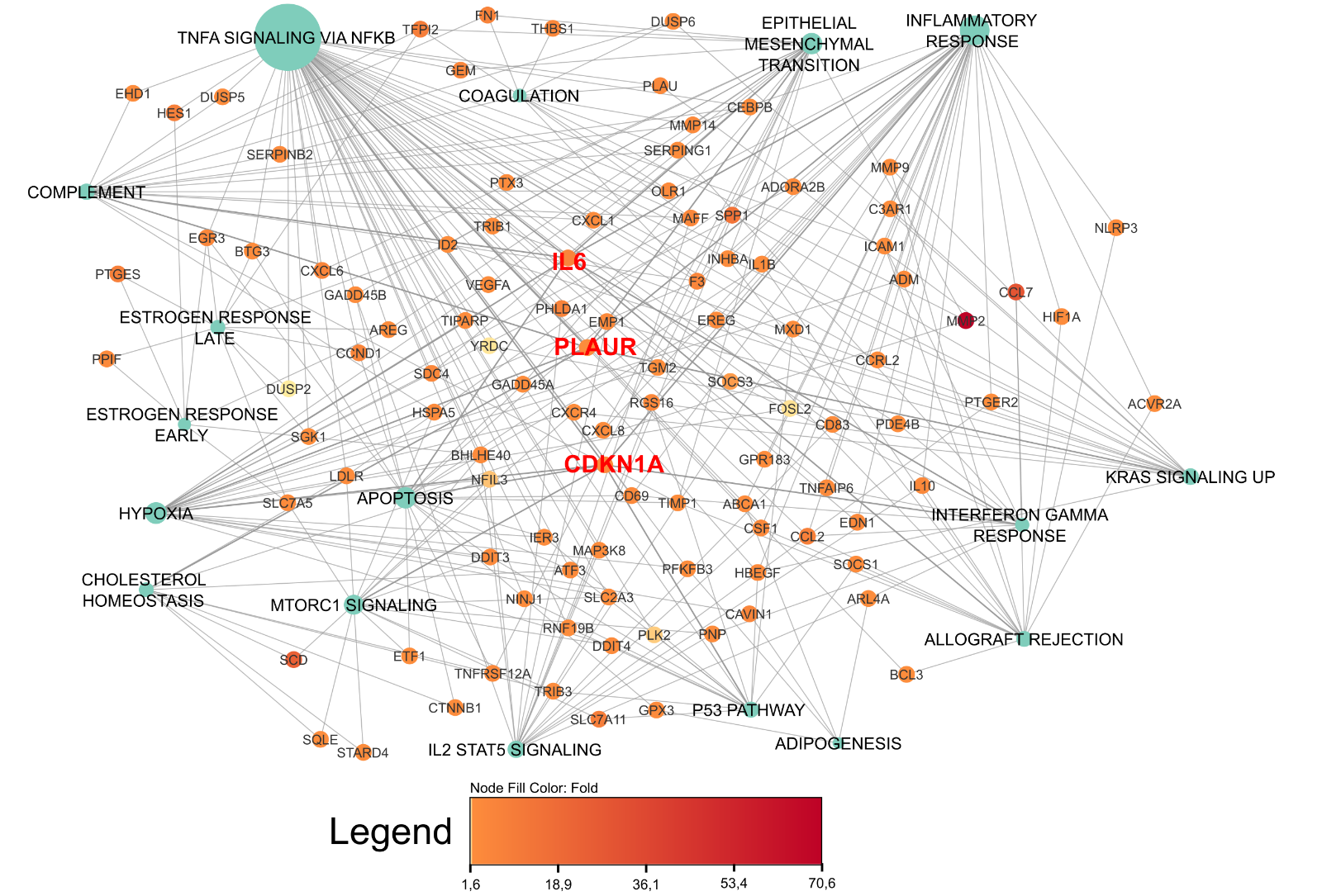


**Supplementary Figure S4.** Highly activated pathways in classical monocytes of bone erosion patients. The most differentially activated pathways are involved in the activation of immune response (A); the positive regulation of leukocyte proliferation (B); and the adaptative immune response (C). ssGSEA scores were compared using Wilcoxon Rank Sum Test. No: patients without erosion; Yes: patients with bone erosion; ssGSEA: single sample Gene Set Enrichment Analysis.


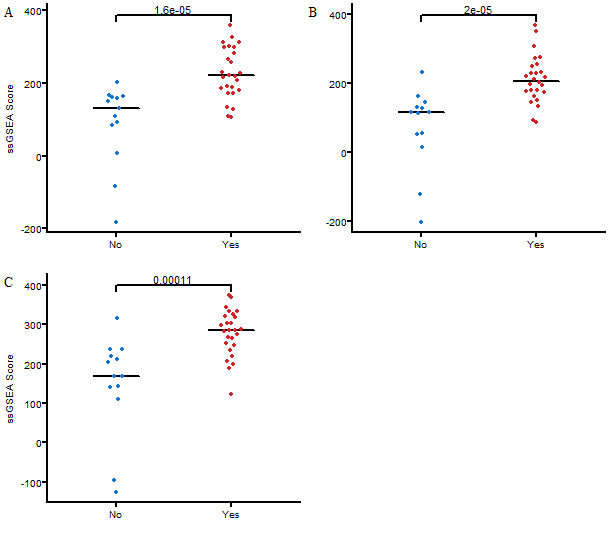


**Supplementary Figure S5.** Classifiers of bone erosion. Using pathway activity at single sample level (ssGSEA) and ROC curve analysis, 3 pathways were identified as good classifiers of bone erosion. These includes: (A) Activation of immune response; (B) Positive regulation of leukocyte proliferation; (C) and the adaptative immune response. ssGSEA: Single Sample Gene Set Enrichment analysis; ROC: Receiver Operating Characteristic.


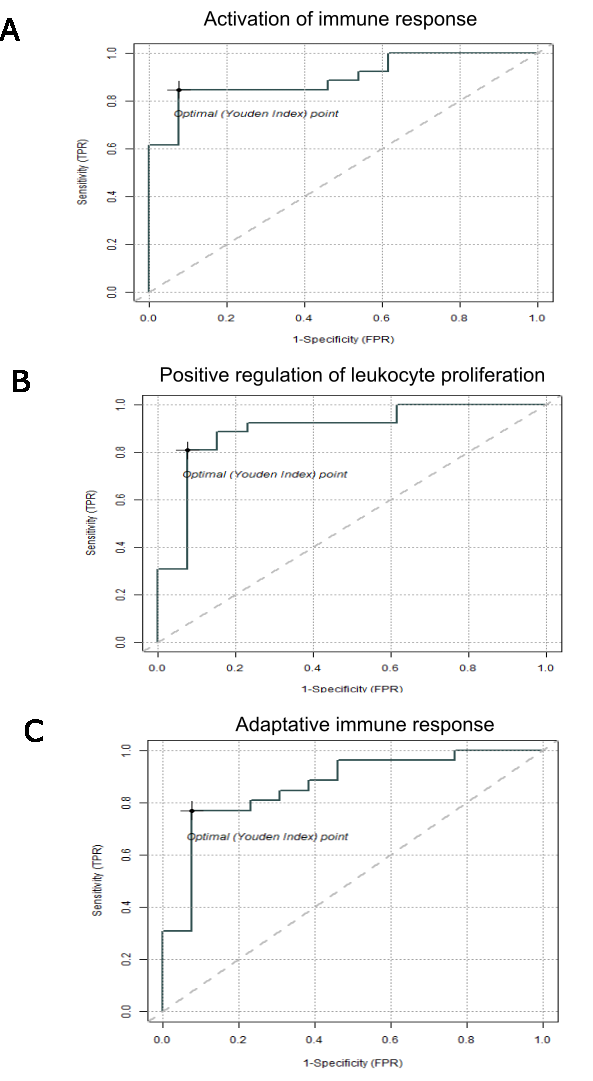

Supplement: Supplementary file 1 [file DataSheet_1.docx]
